# Paroxetine protects against bleomycin-induced pulmonary fibrosis by blocking GRK2/Smad3 pathway

**DOI:** 10.18632/aging.205092

**Published:** 2023-10-09

**Authors:** Kaochang Zhao, Hanxiang Nie, Zheng Tang, Guozhong Chen, Jizhen Huang

**Affiliations:** 1 Department of Respiratory and Critical Care Medicine, Renmin Hospital of Wuhan University, Wuhan 430060, Hubei Province, China; 2 Department of Thoracic Surgery, Zhongnan Hospital of Wuhan University, Wuhan 430060, Hubei Province, China

**Keywords:** pulmonary fibrosis, paroxetine, GRK2, Smad3, lung fibroblasts

## Abstract

G protein-coupled receptor kinase-2 (GRK2) is involved in TGF-β1-induced activation of lung fibroblasts, which could give rise to the pathogenesis of pulmonary fibrosis. Paroxetine (PRXT) serves as a selective GRK2 inhibitor which is widely used to treat anxiety and depression for several decades. However, whether PRXT could inhibit TGF-β1-induced activation of lung fibroblasts and combat bleomycin-induced pulmonary fibrosis remains unclear. Here, we investigated the effects of PRXT on pulmonary fibrosis in C57/BL6 caused by bleomycin as well as on the activation of murine primary lung fibroblasts stimulated with TGF-β1. The results demonstrated that PRXT markedly improved the pulmonary function and 21-day survival in bleomycin-induced mice. Meanwhile, PRXT significantly decreased collagen deposition, inflammation, and oxidative stress in lung tissues from bleomycin-induced mice. Furthermore, we found that PRXT could inhibit the protein and mRNA expression of GRK2 and Smad3 in lung tissues from bleomycin-induced mice. *In vitro* experiments also PRXT could inhibit cell activation and collagen synthesis in a concentration-dependent manner in TGF-β1-induced lung fibroblasts. In addition, we found that Smad3 overexpression by adenovirus transfection could offset anti-fibrotic and antioxidative effects from PRXT in TGF-β1-induced lung fibroblasts, which showed no effects on the protein expression of GRK2. In conclusion, PRXT mediates the inhibition of GRK2, which further blocks the transcription of Smad3 in TGF-β1-induced lung fibroblasts, providing an attractive therapeutic target for pulmonary fibrosis.

## INTRODUCTION

Pulmonary fibrosis is a special form of idiopathic interstitial pneumonia with a high mortality rate as well as an average of two to three years lifespan once it is diagnosed. Pulmonary fibrosis is distinguished by decreased functional capacity, hypoxia, and dyspnea triggered by exercise or at rest. The pathological features of pulmonary fibrosis mainly involve increased collagen deposition as well as collapse of lung structure. During pulmonary fibrosis, impaired lung architecture can irreversibly and progressively affect gas exchange, eventually giving rise to hypoxic respiratory failure. At present in clinical practice, there is still a lack of effective drugs or strategies against pulmonary fibrosis other than lung transplantation. Hence, it is of great significance to search for new drugs or chemical components that could retard or reverse the development of pulmonary fibrosis.

Accumulating evidence demonstrates that the activation of fibroblasts and deposition of the extracellular matrix (ECM) are two direct factors driving the destruction of lung tissue. In lung tissue, fibroblasts could synthesize and secrete proteins, resulting in the formation of the ECM. Under physiological states, the ECM helps to preserve the normal scaffold of lung endothelium and epithelium, playing critical roles in gas exchange and damaged tissue repair. In the context of pro-fibrotic factors stimulation, activated lung fibroblasts could differentiate into myofibroblasts, promoting the synthesis of ECM. In addition, the level of oxidative stress is elevated in lung fibroblasts during pulmonary fibrosis. Hence, proteins or drugs which could inhibit oxidative stress have the potential to alleviate pulmonary fibrosis [1]. For example, mitochondria-targeted reactive oxygen (ROS) scavenger mitoquinone or SIRT1 activator could significantly suppress the activation of lung fibroblasts by decreasing mitochondrial oxidative stress [2]. G protein-coupled receptor kinase-2 (GRK2) is one of the members belonging to the serine/tyrosine-protein kinase family. The main function of GRK2 is to phosphorylate G protein coupled receptors on cell membrane, thus turning off their intracellular signaling under both physiological states and pathological conditions. Yanhui Li et al. reported that GRK2 could promote the activation of lung fibroblast cells by increasing Smad3 expression during pulmonary fibrosis [3]. Also, rat cardiac fibroblasts induced by arginine vasopressin, GRK2 contributed to its proliferation through β-arrestin/ERK1/2 signalling pathway [4]. In diabetic male mice, GRK2 inhibitor significantly relieved the severity of diabetic cardiomyopathy by decreasing inflammation and oxidative stress [5]. These studies suggested that GRK2 may serve as a novel target in blocking the activation of lung fibroblasts and treating pulmonary fibrosis.

Paroxetine (PRXT) is a prescribed Food and Drug Administration (FDA)-approved selective serotonin reuptake inhibitor, which is mainly used to treat depression and anxiety. In recent years, PRXT was also verified as a direct inhibitor of GRK2, which could block the desensitization of GPCR through binding to the active site of GRK2 [6]. It is extensively reported that PRXT exerts important protective effects on cardiac fibrosis. For example, in the context of hypertension, PRXT could decrease cardiac hypertrophy, dysfunction, as well as fibrosis via blocking GRK2-mediated ADRB1 activation [7]. In Wistar rats with myocardial infarction, PRXT treatment significantly relieved left ventricle remodeling as well as susceptibility to arrhythmias by reducing ROS [8]. Based on these findings, we hypothesized that PRXT could effectively protect against bleomycin-induced pulmonary fibrosis by decreasing oxidative stress and blocking the activation of lung fibroblasts through regulating GRK2.

The current research explored the protective effects of PRXT against bleomycin-induced pulmonary fibrosis in mice, with a focus on its effect on oxidative stress and the activation of lung fibroblasts.

## MATERIALS AND METHODS

### Drugs and reagents

Paroxetine (#HY-122272) and bleomycin (#HY-17565A) were obtained from MedChemExpress (MCE) Co. Ltd (Shanghai, China). Transforming growth factor-β1 (TGF-β1) (#SAB4502954) was purchased from Sigma-Aldrich (St. Louis, MO, USA). Primary antibodies including Collagen I (#ab138492), α-SMA (#ab5831), GAPDH (#ab8245), GRK2 (#ab227825), P-Smad3 (#ab52903), Smad3 (#ab40854), P-Smad2 (#ab280888), and Smad2 (#ab40855) were provided by Abcam (Cambridge, United Kingdom). P-Smad3 (#10231-1-AP) was purchased from Proteintech Group, Inc (Rosemont, USA). The BCA assay kit was obtained from Pierce (Rockford, IL, USA).

### Animals and treatment

C57/BL6 male mice (6-8 weeks) purchased from Experimental Animal Research Center, Hubei Center for Disease Control and Prevention were fed in a specific pathogen-free (SPF) room at the center. The laboratory was maintained under controlled temperatures (23° C) and a relative humidity of 46% with a 12 hour light/dark cycle.

**Figure 1:**
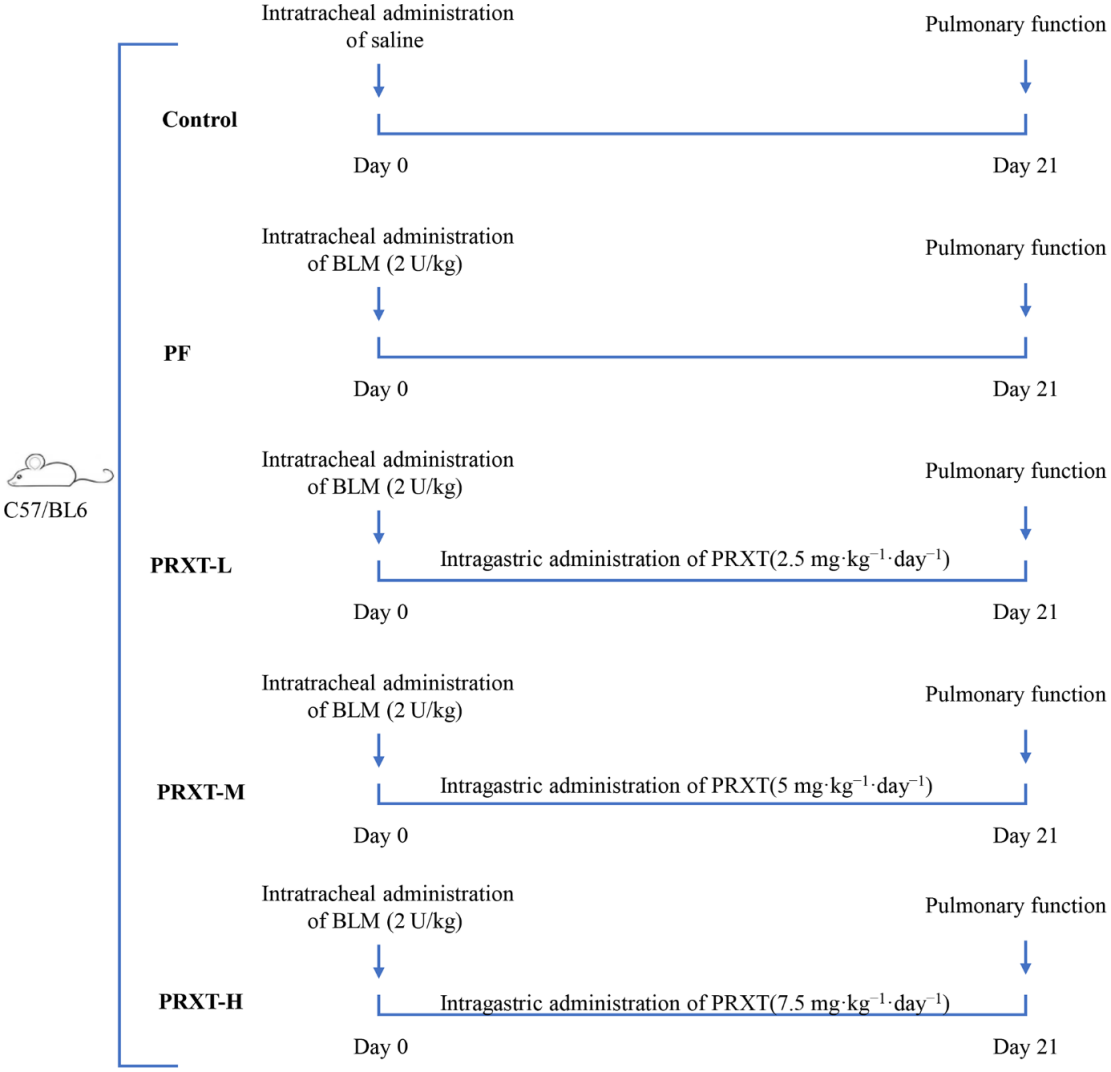
Flow chart presenting the experimental protocol of *in vivo* study.

Mice were randomly divided into 5 groups, the control group (saline), PF group (2 U/kg bleomycin), Paroxetine-Low (PRXT-L) group (2.5 mg·kg^−1^·day^−1^), PRTX-M (5 mg·kg^−1^·day^−1^), and PRTX-H (7.5 mg·kg^−1^·day^−1^). To mimic a murine pulmonary fibrosis model, mice were subjected to intratracheal administration of bleomycin (2 U/kg) after anesthetized with pentobarbital sodium (1%) on the basis of previous description [9]. The design scheme of the present experiment is displayed in Figure 1. From the beginning of bleomycin administration, different dosages of PRXT were administered intragastrically for 21 consecutive days. At the end of the study, pulmonary function and arterial blood gas were analyzed before lung tissues were harvested and weighed.

### The detection of pulmonary function and arterial blood gas

The pulmonary function of mice in the indicated groups was detected on the day 21^st^ using the FinePointe non-invasive testing system (FinePointe™ NAM, Data Sciences International, St. Paul, MN, USA). In detail, mice were fixed in a closed box equipped with an airflow monitoring apparatus on one end of the box. The non-invasive pulmonary function parameters including specific airway resistance (sRaw), specific airway conductance (sGaw), functional residual capacity (FRC), and minute volume (MV) were calculated from the airflow. Meanwhile, arterial blood was also analyzed via an automatic blood gas analyzer (ABL80, Radiometer, Copenhagen, Denmark) once it was collected from left ventricle puncture. The status of acid-base equilibrium of arterial blood gas was reflected by arterial oxygen partial pressure (PaO_2_), arterial carbon dioxide partial pressure (PaCO_2_), and sodium bicarbonate (HCO_3_^-^).

### Cell culture and treatment

Primary murine lung fibroblasts from C57/BL6 mice (8 weeks) were isolated through collagenase-trypsin-DNase digestion according to previous description [10]. Lung fibroblasts seeded in 6-well cell culture dishes were cultured in Dulbecco’s modified Eagle medium (DMEM) supplemented with 10% fetal bovine serum (FBS), 100 μg/ml streptomycin, as well as 100 U/ml penicillin. Once the lung fibroblasts were passaged consecutively to the 3rd to 6th generations, they could be used for subsequent *in vitro* experiments. To induce the activation of lung fibroblasts, 10 ng/mL TGF-β1 was used to incubate for 48 hours. Meanwhile, different concentrations of PRXT (ranging from 0 to 50μM) were added to observe its effects on the activation of lung fibroblasts. Replication-defective adenoviral (Ad) vectors were employed to overexpress Smad3 in lung fibroblasts. In brief, lung fibroblasts were infected with *Ad*-Smad3 diluted in DMEM at 40 MOI. After infection for 24 hours, lung fibroblasts were starved with medium without FBS for 16 hours. After that, TGF-β1 and PRTX were added in medium.

### H&E staining

Cleaned lung tissue specimens were fixed in 4% paraformaldehyde, followed by paraffin embedding and slicing into 4-μm-thick sections. Then the lung tissue sections were dewaxed and then rehydrated. The specimens were then stained with hematoxylin and eosin according to the reagent instructions. The staining of the sections was observed by light microscopy.

### Masson staining

Lung tissue samples were first washed in PBS and then fixed in 4% paraformaldehyde. Paraffin-embedded specimens were sliced into 4-μm-thick section, which were then dewaxed and re-hydrated. Next, the lung tissue sections were stained with three stains according to reagent instructions, namely Hematoxylin, Ponceau S and Aniline Blue. Finally, images were captured through a light microscopy.

### Reverse transcription-polymerase chain reaction (RT-PCR)

Total RNA was first extracted from the tissue by the method trizol and tested for RNA purity and integrity. The extracted RNA was then reverse transcribed to produce cDNA according to the instructions of the corresponding kit. Using Light Cycler 480 SYBR Green Master Mix, the target fragment selected by the synthetic primers was amplified using the cDNA as a template, catalyzed by DNA polymerase, and normalized to *Gapdh*. PCR protocol comprises the following parts: an initial denaturation at 95° C for 5 minutes, followed by 30 cycles of 95° C for 20 seconds, 60° C for 25 seconds and 72° C for 45 seconds. Data were analyzed using the 2^-ΔΔcq^ method. Primer sets are provided in the Supplementary Table 1.

### Western blot

Tissues and cells were harvested, lysed in RIPA buffer containing protease inhibitor cocktail and phosphatase inhibitor cocktail, sonicated and precipitated by centrifugation at 12,000 x g for 30 minutes at 4° C. The concentrations of protein were assessed using a bicinchoninic acid (BCA) protein assay kit. Protein samples (35 μg per lane) were separated on 10%-12% SDS-PAGE gels and transferred to PVDF membranes and blocked with 5% skimmed milk for 1 hour at room temperature. The membrane was then incubated with the appropriate primary and secondary antibodies at the appropriate level. The primary antibodies as well as their dilution ratio are shown as follows: Collagen I (1:1000), α-SMA (1:1000), GRK2 (1:500), GAPDH (1:1000), phosphorylated-Smad3 (p-Smad3) (1:500), Smad3 (1:500), p-Smad2 (1:500), Smad2 (1:1000), and Smad4 (1:500). The relative protein levels were normalized with the level of GAPDH. The membranes were scanned using an Odyssey infrared imaging system and the results were identified using Image J software.

### Determination of oxidative stress

SOD activity, NADPH oxidase activity and MDA level in lung tissue samples and lung fibroblast were detected via commercial kits on the basis of the manufacturer's instructions.

### Cell counting kit-8 (CCK-8)

A CCK-8 assay kit was employed to measure the cell viability of treated lung fibroblasts. A 100-ml cell suspension was isolated and added into a 96-well plate. Then the cell viability was detected using a commercial assay kit (#ab228554, Abcam) according to the instruction.

### Data analysis

A log-rank test was used to draw survival curves. All data in our study are presented as mean ± standard deviation (SD) for the number (n) of experiments, which was analyzed using the software SPSS 23.3. Differences among three groups were analyzed by one way ANOVA with Tukey post hoc test while differences between two groups were analyzed by student’s unpaired t-test. Statistical difference was confirmed with *P < 0.05*.

### Data availability

The data that support the findings of this study are available from the corresponding author upon reasonable request.

## RESULTS

### PRXT treatment preserved pulmonary function in bleomycin-induced mice

**Figure 2:**
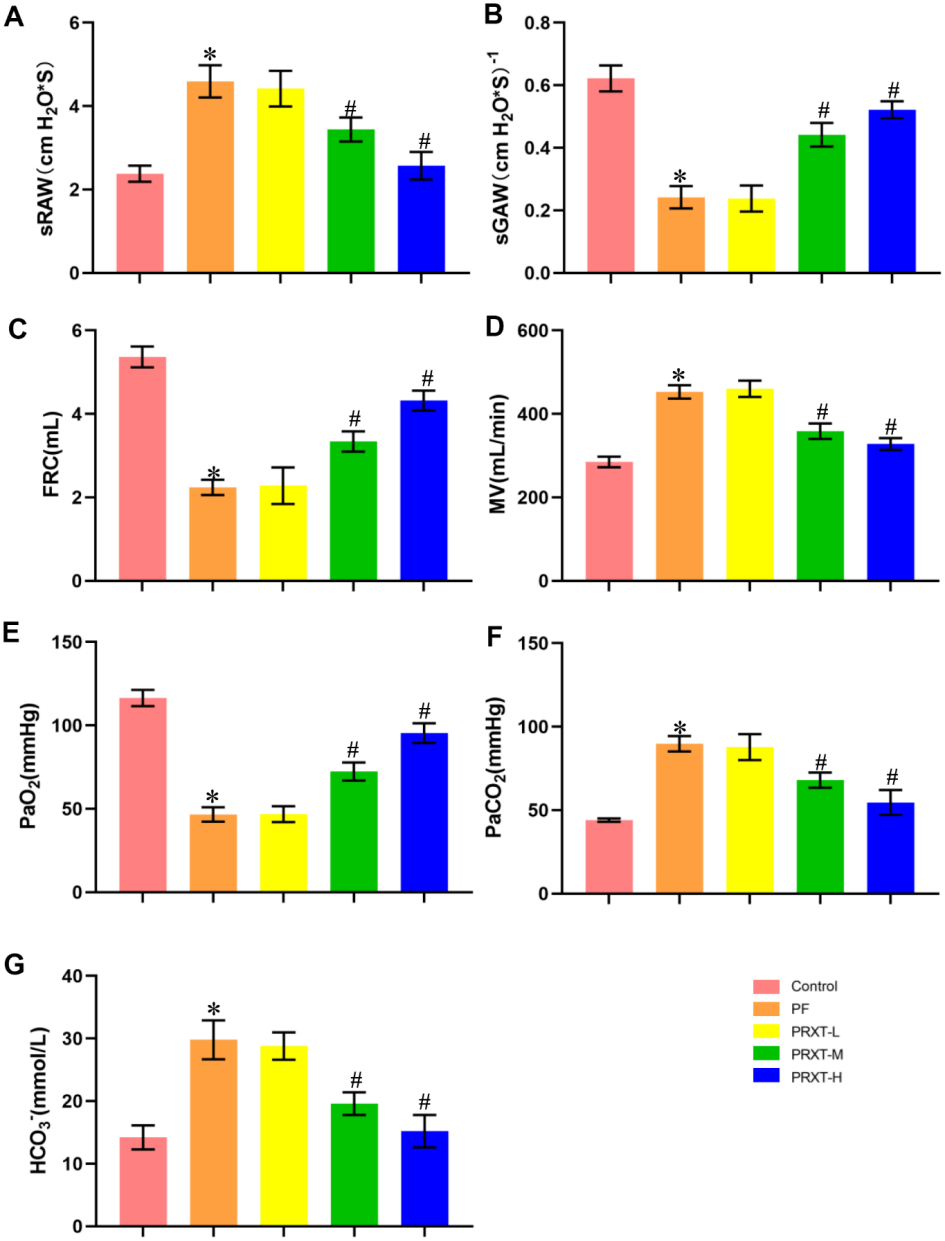
**PRXT treatment ameliorated lung pathological injury and improved survival rate in bleomycin-induced mice.** (**A**–**D**) Analyses of pulmonary function parameters in the indicated groups including specific airway resistance (sRaw), specific airway conductance (sGaw), functional residual capacity (FRC), as well as minute volume (MV) (n=5). (**E**–**G**) Analyses of arterial blood gas including PaO_2_, PaCO_2_, and HCO_3_^-^ in the indicated groups (n=5). *P < 0.05 vs. the Control group, ^#^*P* < 0.05 vs. the PF group. PF, pulmonary fibrosis. PRTX-L, Paroxetine-Low dose (2.5 mg·kg^−1^·day^−1^). PRTX-M, Paroxetine-Medium dose (5 mg·kg^−1^·day^−1^). PRTX-H, Paroxetine-High dose (7.5 mg·kg^−1^·day^−1^).

Mice injected with bleomycin (2 U/kg) presented with symptoms of pulmonary dysfunction evidenced by increased sRAW, decreased sGAW, reduced FRC, and increased MV in PF group compared with the Control group (Figure 2A–2D). Pulmonary function in PRXT-M group and PRXT-H group was significantly improved compared with that in the Control group, indicating that PRXT administration (5 mg·kg^−1^·day^−1^ and 7.5 mg·kg^−1^·day^−1^) attenuated bleomycin-induced pulmonary dysfunction. However, the lower PRXT dose (2.55 mg·kg^−1^·day^−1^) did not significantly prevent bleomycin-induced pulmonary dysfunction compared with the PF group (Figure 2A–2D). Arterial blood gas was further analyzed to examine pulmonary function and acid-base equilibrium. The results showed that PRXT administration (5 mg·kg^−1^·day^−1^ and 7.5 mg·kg^−1^·day^−1^) remarkedly restored PaO_2_, PaCO_2_, and HCO_3_^-^ in bleomycin-induced mice while the lower PRXT dose (2.5 mg·kg^−1^·day^−1^) did not affect the status of arterial blood gas compared with the PF group (Figure 2E–2G). Taken together, these data demonstrated that PRTX administration ameliorated bleomycin-induced pulmonary dysfunction and preserved acid-base equilibrium of arterial blood.

### PRXT treatment ameliorated lung pathological injury and improved survival rate in bleomycin-induced mice

**Figure 3:**
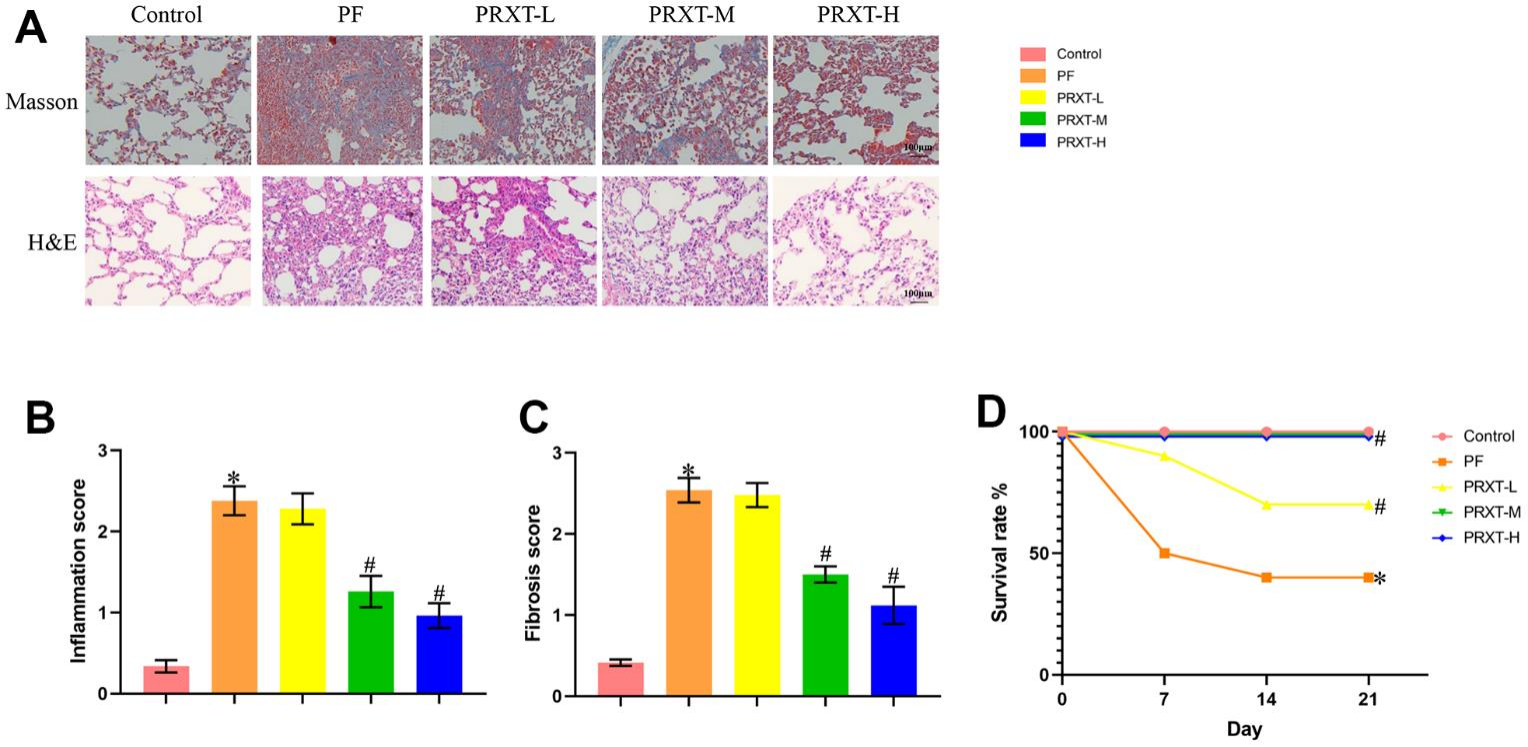
**PRXT treatment ameliorated lung pathological injury and improved survival rate in bleomycin-induced mice.** (**A**) Representative images of H&E staining and Masson staining in the indicated groups (n=5). (**B**, **C**) Inflammation score and fibrosis score based on H&E staining and Masson staining (n=5). (**D**) Survival rate of each group (n=10). *P < 0.05 vs. the Control group, ^#^*P* < 0.05 vs. the PF group. PF, pulmonary fibrosis. PRTX-L, Paroxetine-Low dose (2.5 mg·kg^−1^·day^−1^). PRTX-M, Paroxetine-Medium dose (5 mg·kg^−1^·day^−1^). PRTX-H, Paroxetine-High dose (7.5 mg·kg^−1^·day^−1^).

H&E staining and Masson staining (Figure 3A) showed that bleomycin stimulation significantly promoted inflammatory response and collagen deposition in lung tissues, evidenced by higher inflammation score and fibrosis score compared with Control group (Figure 3B, 3C). PRXT administration (5 mg·kg^−1^·day^−1^ and 7.5 mg·kg^−1^·day^−1^) remarkedly inhibited inflammation score and fibrosis score in lung tissues from bleomycin-induced mice. In addition, we observed the effects of PRXT administration on 21-day survival rate in bleomycin-induced mice. The data showed that bleomycin stimulation remarkedly decreased survival rate in mice while PRXT administration (5 mg·kg^−1^·day^−1^ and 7.5 mg·kg^−1^·day^−1^) could restored 21-day survival rate in bleomycin-induced mice. Similarly, the lower PRXT dose (2.5 mg·kg^−1^·day^−1^) did not affect the survival rate in bleomycin-induced mice compared with the PF group (Figure 3D). These results indicated that PRXT treatment effectively reduced ameliorated lung pathological injury and improved survival rate in bleomycin-induced mice.

### PRXT treatment suppressed collagen synthesis, inflammation and oxidative stress in bleomycin-induced mice

**Figure 4:**
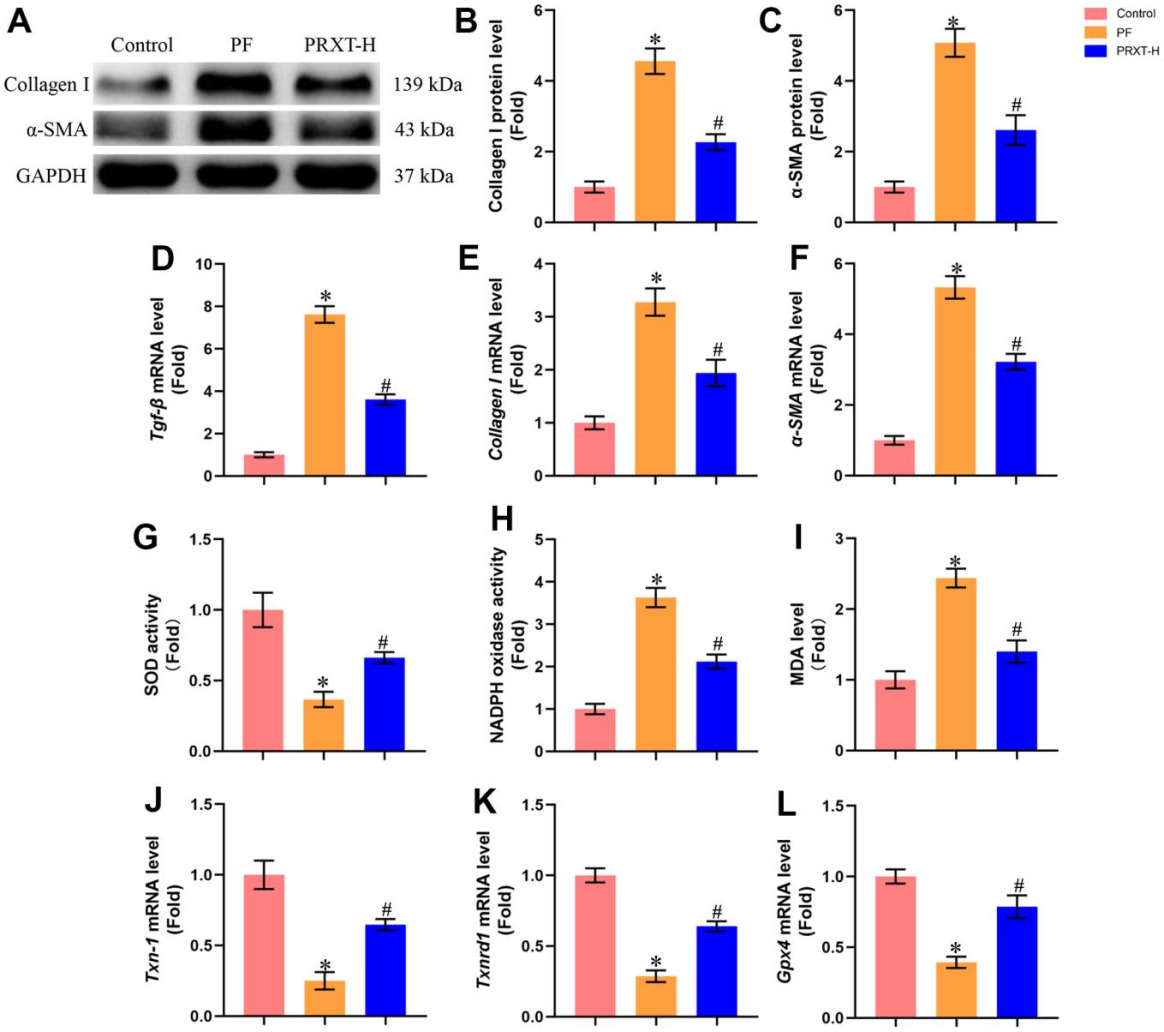
**PRXT treatment suppressed collagen synthesis, inflammation and oxidative stress in bleomycin-induced mice.** (**A**–**C**) Representative western blotting images of Collagen I and α-SMA and their quantification in murine lung tissues (n=5). (**D**–**F**) Relative mRNA expression levels of Tgf-β, Collagen I and α-SMA in murine lung tissues (n=5). (**G**) SOD activity (n=5). (**H**) NADPH oxidase activity (n=5). (**I**) MDA level (n=5). (**J**–**L**) Relative mRNA expression levels of Txn-1, Txnrd1, and Gpx4 in murine lung tissues (n=5). *P < 0.05 vs. the Control group, ^#^*P* < 0.05 vs. the PF group. PF, pulmonary fibrosis. PRTX-H, Paroxetine-High dose (7.5 mg·kg^−1^·day^−1^).

Western blot (Figure 4A–4C) showed that bleomycin stimulation significantly upregulated the protein levels of Collagen I and α-SMA in murine lung tissues. PRXT administration (7.5 mg·kg^−1^·day^−1^) could inhibit the protein expression of Collagen I and α-SMA compared with PF group. RT-PCR (Figure 4D–4F) also unveiled that the mRNA levels of Tgf-β, Collagen I, and α-SMA in lung tissues were upregulated by bleomycin stimulation, which could be restored by PRXT administration (7.5 mg·kg^−1^·day^−1^). Also, we measured oxidative stress levels in the indicated groups. Bleomycin stimulation could decrease SOD activity and enhance NADPH oxidase activity in lung tissues, apart from upregulating MDA levels (Figure 4G–4I). RT-PCR also showed that bleomycin stimulation could significantly downregulate the mRNA levels of *Txn-1*, *Txnrd1*, and *Gpx4* in lung tissue (Figure 4J–4L). As expected, PRXT administration (7.5 mg·kg^−1^·day^−1^) could inhibit oxidative stress in lung tissues compared with PF group. Also, we accessed the effects of PRTX-H treatment on hepatic and renal function in mice. As shown in Supplementary Figure 1A, 1B, PRTX-H treatment for 21 days showed no effects on serum creatinine and serum ALT, suggesting the therapeutic dose of PRTX displayed no hepatotoxicity and renal toxicity. Taken together, these results suggested PRXT treatment suppressed collagen synthesis, inflammation and oxidative stress in mice with PF.

### PRXT promoted the expression of GRK2 and Smad3 in lung tissues from bleomycin-induced mice

**Figure 5:**
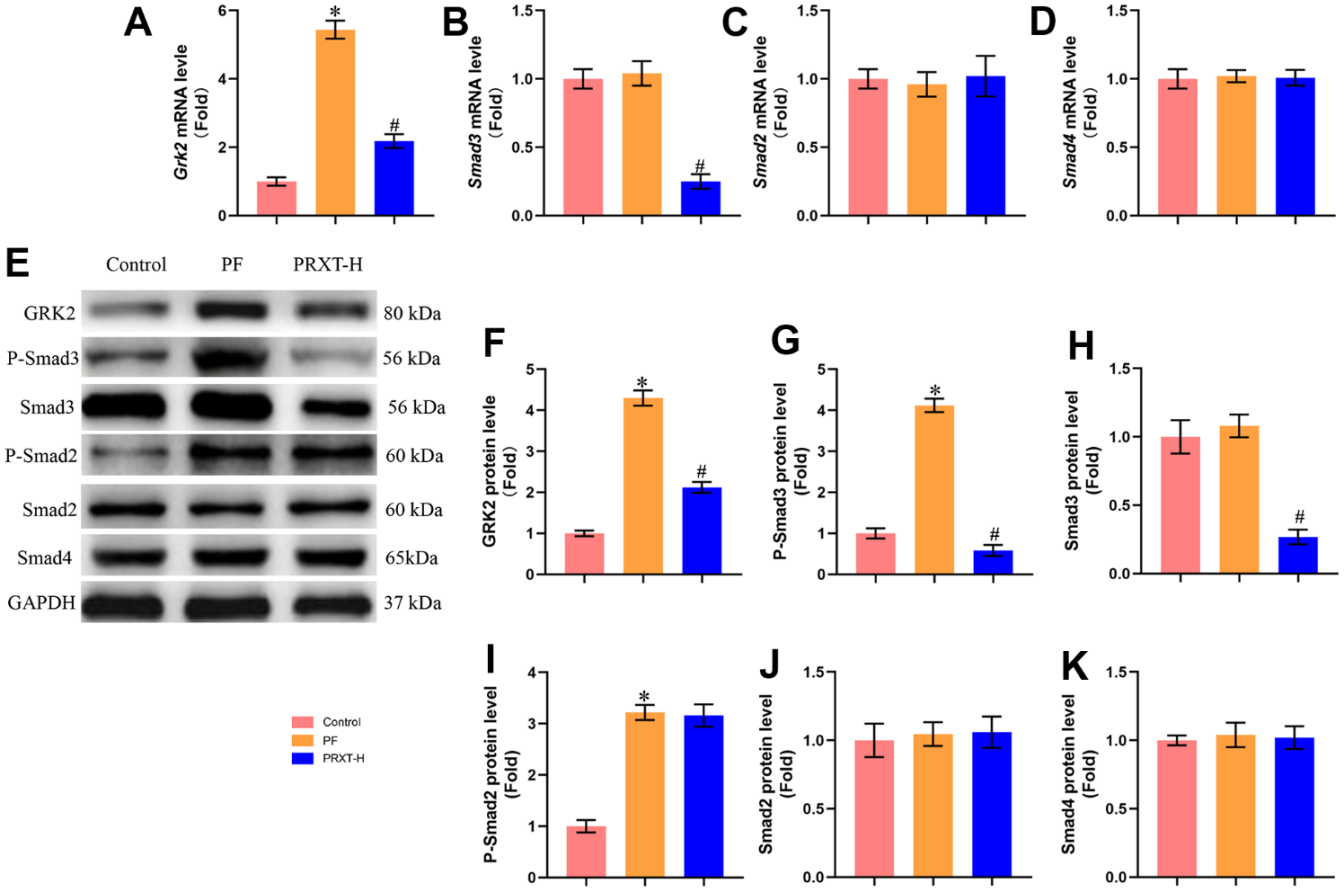
**PRXT promoted the expression of GRK2 and Smad3 in lung tissues from bleomycin-induced mice.** (**A**–**D**) Relative mRNA expression levels of Grk2, Smad3, Smad2, and Smad4 in murine lung tissues (n=5). (**E**–**K**) Representative western blotting images of GRK2, P-Smad3, Smad3, P-Smad2, Smad2, and Smad4 and their quantification in murine lung tissues (n=5). *P < 0.05 vs. the Control group, ^#^*P* < 0.05 vs. the PF group. PF, pulmonary fibrosis. PRTX-H, Paroxetine-High dose (7.5 mg·kg^−1^·day^−1^).

One previous study has reported that GRK2 levels were increased in lungs and isolated fibroblast cells during pulmonary fibrosis and GRK2 inhibition could decrease extracellular matrix (ECM) accumulation by downregulating Smad3 expression [3]. Herein, we next detected the expression of GRK2 and TGF-β/Smads pathway. RT-PCR showed that bleomycin stimulation significantly upregulated the mRNA expression of Grk2. PRXT administration (7.5 mg·kg^−1^·day^−1^) could inhibit the mRNA level of Grk2 in lung tissues from bleomycin-induced mice (Figure 5A). Meanwhile, we detected the mRNA levels of Smad3, Smad2, and Smad4. The data showed that bleomycin stimulation showed no effects on the mRNA levels of Smad3, Smad2, and Smad4 in lung tissues from bleomycin-induced mice compared with Control group (Figure 5B–5D). PRXT administration (7.5 mg·kg^−1^·day^−1^) significantly inhibited the mRNA level of Smad3 but showed no effects on the mRNA levels of Smad2 and Smad4. Western blot further showed that bleomycin stimulation significantly upregulated the protein levels of GRK2, P-Smad3 and P-Smad2 but displayed no effects on the protein levels of Smad3, Smad2, and Smad4 (Figure 5E–5K). Intriguingly, PRXT administration (7.5 mg·kg^−1^·day^−1^) significantly downregulated the protein levels of GRK2, P-Smad3 and Smad3 in lung tissues from bleomycin-induced mice, suggesting that the inhibitory effects of PRXT on P-Smad3 may be implicated with the decrease of total Smad3 in bleomycin-induced lung tissues. Collectively, these data further unveiled that PRXT-mediated inhibition of GRK2 decreased the mRNA and protein expression of Smad3 during pulmonary fibrosis.

### PRXT inhibited TGF-β1-induced activation of lung fibroblasts in a concentration-dependent manner

**Figure 6:**
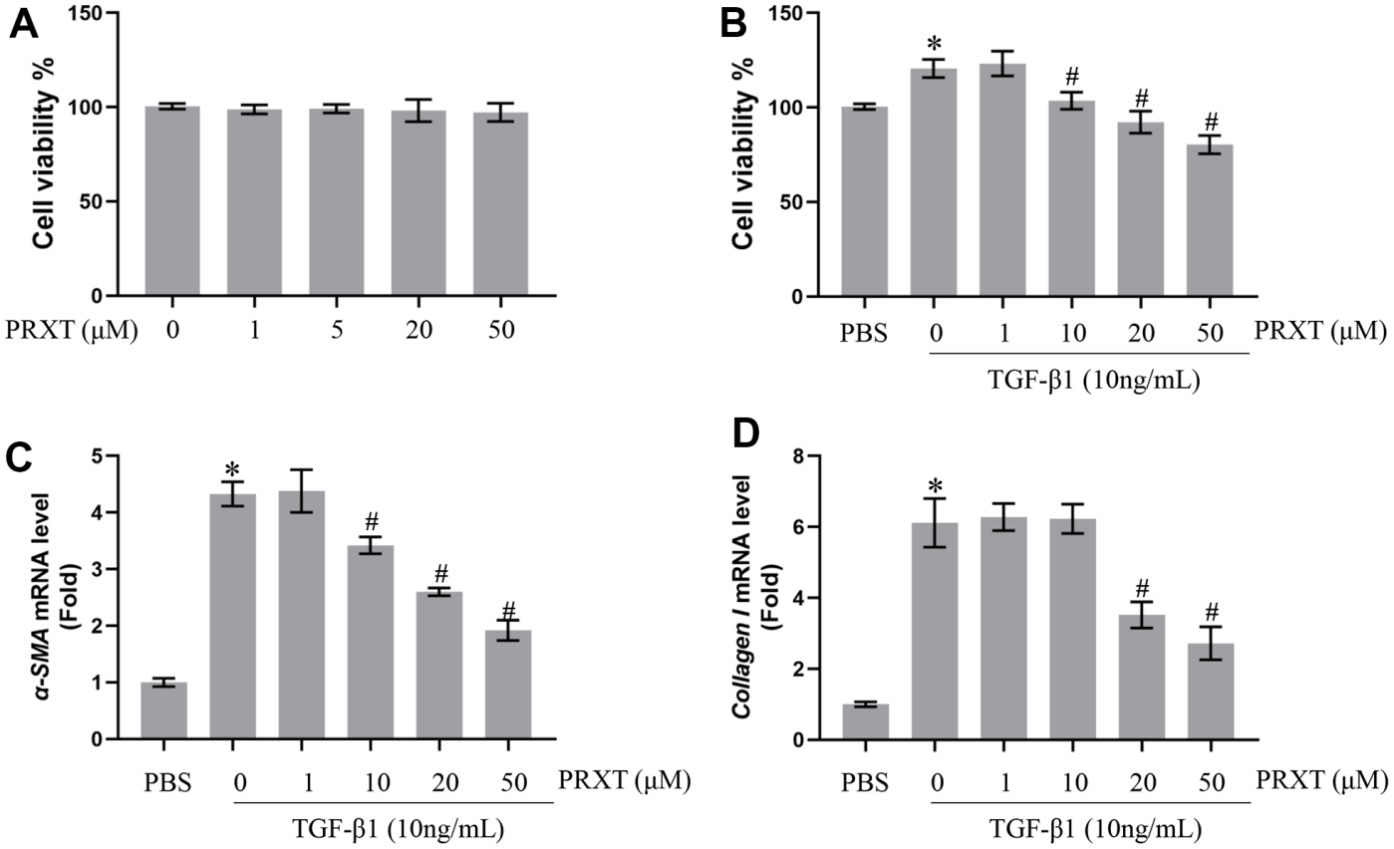
**PRXT inhibited TGF-β1-induced activation of lung fibroblasts in a concentration-dependent manner.** (**A**) Cell viability in primary lung fibroblasts treated with different concentrations of PRTX (n=5). (**B**) Cell viability in TGF-β1-induced primary lung fibroblasts treated with different concentrations of PRTX (n=5). (**C**, **D**) Relative mRNA expression levels of α-SMA and Collagen I in primary lung fibroblasts (n=5). *P < 0.05 vs. the PBS group, ^#^*P* < 0.05 vs. the TGF-β1 group. PRTX, Paroxetine.

Next, we observed the protective effects of PRXT on TGF-β1-induced lung fibroblasts. Figure 6A showed that PRXT (ranging from 0 to 50 μM) did not affect cell viability of primary lung fibroblasts. In TGF-β1 (10 ng/mL)-induced lung fibroblasts, 10, 20 and 50 μM of PRXT could significantly inhibit the cell viability and the mRNA level of α-SMA (Figure 6B, 6C). And 20 and 50 μM of PRXT also decreased the mRNA level of Collagen I in TGF-β1-induced lung fibroblasts (Figure 6D). Taken together, these data disclosed that PRXT inhibited TGF-β1-induced activation of lung fibroblast in a concentration-dependent manner.

### Smad3 overexpression offset PRXT-elicited protection against TGF-β1-induced activation of lung fibroblasts

**Figure 7:**
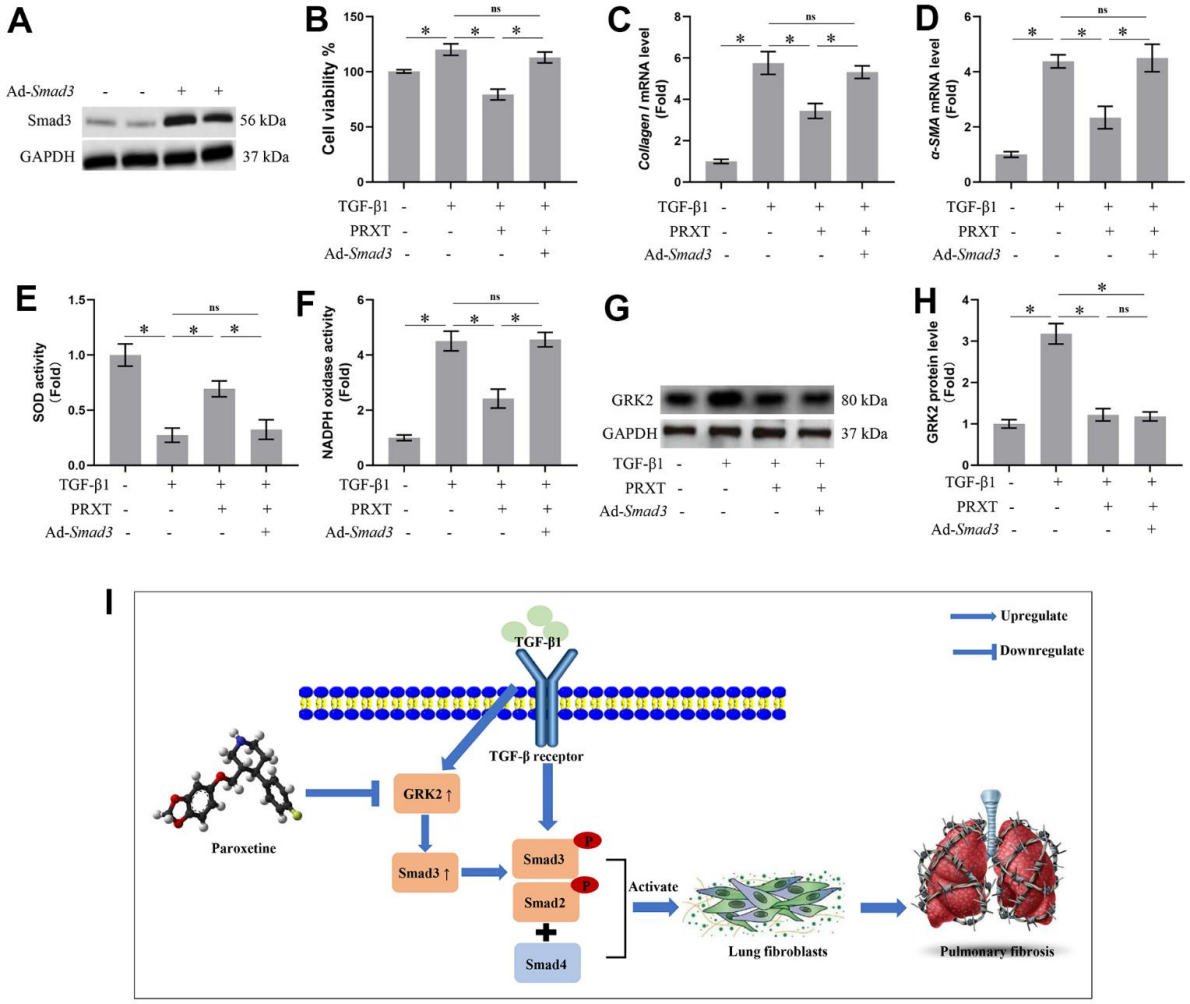
**Smad3 overexpression offset PRXT-elicited protection against TGF-β1-induced activation of lung fibroblasts.** (**A**) Representative western blotting images of Smad3 in primary lung fibroblasts transfected with Ad-*Smad3*. (**B**) Cell viability in the indicated groups (n=5). (**C**, **D**) Relative mRNA expression levels of Collagen I and α-SMA in the indicated groups (n=5). (**E**, **F**) SOD activity and NADPH oxidase activity (n=5). (**G**, **H**) Representative western blotting images of GRK2 and its quantification in the indicated groups (n=5). (**I**) Chemical structure of PRXT and the possible mechanisms of the protective effects of PRXT in pulmonary fibrosis. Arrowheads denote the upregulate while the “T” ending lines represent downregulate.

To further confirm Smad3 was involved in PRXT-elicited protection against TGF-β1-induced activation of lung fibroblast, we used adenovirus to overexpress the expression of Smad3 in lung fibroblasts (Figure 7A). In this part, we observed the role of PRXT (50 μM) on lung fibroblast stimulated with TGF-β1 for 49 hours. As expected, PRXT (50 μM) treatment significantly inhibited the activation of lung fibroblasts and the mRNA levels of Collagen I and α-SMA. By contrast, Smad overexpression could restore the cell viability and collagen synthesis in TGF-β1-induced lung fibroblasts. We also found that the cell viability and collagen synthesis in TGF-β1 group showed no difference compared with TGF-β1-PRXT-Ad-*Smad3* group (Figure 7B–7D). Also, we detected the oxidative stress level in the indicated groups. Smad3 overexpression offset antioxidation of PRXT in TGF-β1-induced lung fibroblasts, evidenced by decreased SOD activity and increased NADPH oxidase activity (Figure 7E, 7F). Finally, we also found that Smad3 overexpression showed no effects on the protein level of GRK2 in TGF-β1-induced lung fibroblasts treated with PRXT, indicating that GRK2 served as the upstream protein of Smad3 (Figure 7G, 7H). Collectively, these data further demonstrated that Smad3 overexpression offset PRXT-elicited protection against TGF-β1-induced activation of lung fibroblasts.

## DISCUSSION

Idiopathic pulmonary fibrosis is a prototype of chronic and progressive lung disease, which mainly occurs in occur in middle-aged and elderly people [11]. Pulmonary fibrosis is mainly characterized by fibrosis and honeycomb alterations of the subpleural as well as basement membranes, and the excessive deposition of ECM around the fibrotic foci, which ultimately gives rise to irreversible and life-threatening structural alteration in lung tissue and impairment of pulmonary function [11, 12]. Bleomycin could give rise to severe toxicity effects in lung tissues by inducing remodeling of lung architecture and pulmonary dysfunction, eventually leading to respiratory failure. Hence, at present, bleomycin-induced pulmonary fibrosis has been an ideal preclinical animal model for studying the mechanisms contributing to pulmonary fibrosis or screening potential candidates to combat pulmonary fibrosis [13]. This study demonstrated that PRXT treatment could prevent bleomycin-induced pulmonary fibrosis *in vivo* and *in vitro* by regulating GRK2/Smad3 signaling pathway (Figure 7I). Our study suggested that PRXT may act as a potential therapy or adjuvant therapy for pulmonary fibrosis.

Pulmonary fibrosis is driven by a consequence of multiple interacting genetic and environmental risk factors. Multiple mechanisms contribute to pulmonary fibrosis by affecting collagen synthesis and ECM degradation, including activation of lung fibroblasts [14], macrophage M2 program [15], and epithelial-mesenchymal transition (EMT) [16]. Among these mechanisms, activation of lung fibroblast exerts a central role in mediating pulmonary fibrosis by transdifferentiating into the myofibroblast which serves as a classic pathologic fibroblast phenotype. Myofibroblasts could secrete much more matrix, including Collagen I compared with resident lung fibroblasts. TGF-β1 could activate lung fibroblasts and accelerate the process of transdifferentiation. It is worthy note that the durable myofibroblast phenotype requires ongoing activation by TGF-β1 [17, 18]. Oxidative stress is a strained status caused by an imbalance between the antioxidation and oxidation systems, which is also an important pathogenesis of pulmonary fibrosis. Hence, inhibiting oxidative stress could also delay the activation of lung fibroblasts [19]. Previous studies unveiled that the expression and activity of SOD2, one of the main antioxidant enzymes, was reduced during pulmonary fibrosis. Mitoquinone treatment significantly blocked the activation of lung, cardiac, and nasal fibroblasts [2, 20]. In rheumatoid arthritis fibroblast-like synoviocytes, miR-30a-3p ameliorated oxidative stress via activation of Nrf2-ARE signaling pathway [21]. In addition, activation of ALDH2 in cardiac fibroblasts could significantly relieve high glucose-induced oxidative stress, thus preventing diabetic myocardial fibrosis [22]. In line with these studies, our present study also observed obvious oxidative stress and activation of lung fibroblasts in bleomycin-induced mice and TGF-β1-induced lung fibroblasts. However, PRXT treatment could significantly decreased oxidative stress and activation of lung fibroblasts, thus combating pulmonary fibrosis.

It is reported that GRKs could recognize and phosphorylate agonist-activated G protein-coupled receptors (GPCRs) specifically, giving rise to the uncoupling of heterotrimeric G proteins as well as receptor desensitization [23]. GRKs not only interacted with cellular proteins associated with signal transduction upon GPCR activation but also phosphorylated some certain non-GPCR proteins [24]. GRK2 is one of seven GRKs (GRK1-GRK7), which shared similar domains with other members. GRK2 is ubiquitously expressed throughout the body, serving as a core node in a great many signal transduction pathways and displaying a complicated interactome. By interacting with GPCR and non-GPCR proteins, GRK2 participated in the regulation of various cell behaviors including proliferation, differentiation, migration, and cycle [24–26]. TGF-β1 was a key mediator in the pathogenesis of pulmonary fibrosis through activating the downstream Smad proteins. Once Smad3 and Smad2 were activated by TGF-β1, they can bind to Smad4 and translocate to the nucleus, subsequently directly binding to DNA sequences and activating the fibrotic genes [27]. And Smad3 deficiency relieved bleomycin- or TGF-β1-induced pulmonary fibrosis in mice [27, 28]. GRK2 promoted TGFβ1-induced ECM accumulation by transcriptionally activating Smad3 in lung fibroblasts, the inhibition of which thus attenuated bleomycin-induced lung fibrosis [3]. PRTX is a critical serotonin reuptake inhibitor that is mainly used in the treatment of depression, anxiety, as well as obsessive-compulsive disorder [29]. Recent studies have also reported that PRTX as a GRK2 inhibitor capable of preserving cardiac function and decreasing remodeling in acute myocardial infarction and heart failure [7, 30, 31]. In isoprenaline-induced cardiac fibrosis, the PRTX-treated mice displayed lower mRNA levels of fibrosis markers including *Ctgf* and *Collagen I* in cardiac tissues [7]. In addition, by inhibiting GRK2, PRTX could also prevent pathological chondrocyte hypertrophy and IgE-mediated anaphylaxis [32, 33]. Here, we also found that PRTX could inhibit GRK2 during pulmonary fibrosis, subsequently blocking the activation of TGF-β1/Smad3 pathway. We also found that GRK2 inhibition by PRTX decreased oxidative stress level during pulmonary fibrosis, hinting that GRK2 may regulated the proteins responsible for redox biology. In fact, GRK2 inhibitor could decrease oxidative stress and inhibit the generation of ROS in response to isoprenaline stimulation by decreasing Nox4 expression [34]. However, there are still some limitations in the present study. To begin with, whether PRTX treatment could affect other types of cells which are implicated with pulmonary fibrosis, including macrophages, alveolar epithelial cells, and microvascular endothelial cells. Secondly, how PRTX inhibited the transcription of GRK2 in lung fibroblasts also needs exploring.

In summary, our study indicated that supplementation of PRTX relieved pulmonary dysfunction and fibrosis following bleomycin stimulation *in vivo*, and blocked transdifferentiation of lung fibroblasts *in vitro*. The beneficial effects of PRXT on pulmonary fibrosis may be attributed to its inhibition on GRK2 as well as Smad3 in lung fibroblasts. Our study collectively provided PRTX as a novel candidate in the treatment of pulmonary fibrosis in the future.

## Supplementary Material

Supplementary Figure 1

Supplementary Table 1
