# Supplementary material for: Paroxetine protects against bleomycin-induced pulmonary fibrosis by blocking GRK2/Smad3 pathway
Source: Aging (Albany NY). 2023 Oct 9;15(19):10524–39. doi: 10.18632/aging.205092 (PMC10599755; doi:10.18632/aging.205092)
Supplement: Supplementary Figure 1 [file aging-15-205092-s001.pdf]

SUPPLEMENTARY FIGURE

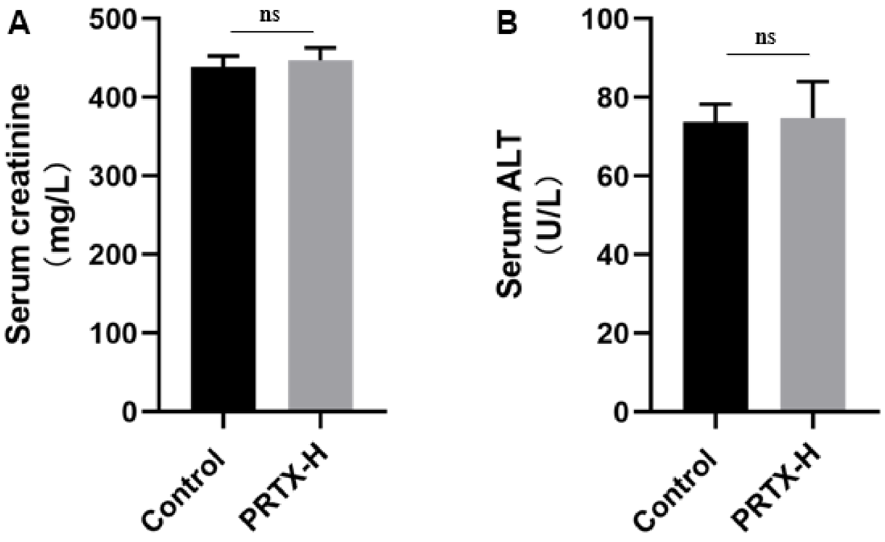

Supplementary Figure 1. The effects of PRTX-H treatment on hepatic and renal function. (**A**) Serum creatinine level in mice, (n=5). (**B**) Serum alanine aminotransferase (ALT) level in mice, (n=5). *P < 0.05 vs. the Control group, PRTX-H, Paroxetine-High dose (7.5 mg·kg^{-1}·day^{-1}).
