# Supplementary material for: Paroxetine protects against bleomycin-induced pulmonary fibrosis by blocking GRK2/Smad3 pathway
Source: Aging (Albany NY). 2023 Oct 9;15(19):10524–39. doi: 10.18632/aging.205092 (PMC10599755; doi:10.18632/aging.205092)
Supplement: Supplementary Table 1 [file aging-15-205092-s002.pdf]

SUPPLEMENTARY TABLE

Supplementary Table 1. The primers used in the study.

Species	Gene	Forward primer	Reverse primer
Mice	*Tgf-β*	ACGTAGTGCTGATGCCAAC	ATGATCGATCGATGATGGTAC
Mice	*Collagen-I*	AATGATGCTAGTACGTACCA	CCCGTAGTAGTCGACCTAGTCAC
Mice	*Collagen-III*	TGGTCGTAGTGTCGTGACCA	AGTCGTAGTCGTGTAACGTAGC
Mice	*a-Sma*	CTGTAGTGTGTCGTACCACC	CCCGTGATGTCGTAGTCGTAGTCGT
Mice	*Grk2*	TGTAGTCGATGTGTAGCACAC	GGTGCTAGTCGATGAACACC
Mice	*Smad2*	TGATCGTACGTAGTGATGCCCA	TCGTCGATCGATCGATCAGCAC
Mice	*Smad3*	CCCCTGATGTCACGTGTAC	TCCATGTGTGCACTTAAGAACCGG
Mice	*Smad4*	ACTGATCGTAGTAGTGTGATGC	TGTCGTGACCCTAGTGATCGAC
Mice	*Txn-1*	CTAGTCGTAGATGCTAAGTCA	CTGATGATGCTGATAGATGCTGA
Mice	*Txnrd1*	AACTGATGTCGTAGTCGTAGA	ACGTAGTCGTAGTGCTGTAAC
Mice	*Gpx4*	ATGTCGTAGTCGTGATGTCGTA	CTAGTCGTAAGATGCTAACCAC
Mice	*Gapdh*	ACTCCTGATGCTAGCTGTGTACA	ACGTGTAGTCGTACCACCCAC
